# *De novo* DNA methylation during monkey pre-implantation embryogenesis

**DOI:** 10.1038/cr.2017.25

**Published:** 2017-02-24

**Authors:** Fei Gao, Yuyu Niu, Yi Eve Sun, Hanlin Lu, Yongchang Chen, Siguang Li, Yu Kang, Yuping Luo, Chenyang Si, Juehua Yu, Chang Li, Nianqin Sun, Wei Si, Hong Wang, Weizhi Ji, Tao Tan

**Affiliations:** 1 Yunnan Key Laboratory of Primate Biomedical Research, Institute of Primate Translational Medicine, Kunming University of Science and Technology, Kunming, Yunnan 650500, China; 2 Agricultural Genomics Institute at Shenzhen, Chinese Academy of Agricultural Sciences, Shenzhen, Guangdong 518120, China; 3 National Engineering Research Center of Biomedicine and Animal Science, Kunming, Yunnan 650500, China; 4 Translational Stem Cell Research Center, Tongji Hospital, Tongji University School of Medicine, Shanghai 200092, China; 5 Department of Psychiatry and Biobehavioral Sciences, UCLA Medical School, Los Angeles, CA 90095, USA

**Keywords:** rhesus monkey, pre-implantation embryogenesis, *de novo* DNA methylation

## Abstract

Critical epigenetic regulation of primate embryogenesis entails DNA methylome changes. Here we report genome-wide composition, patterning, and stage-specific dynamics of DNA methylation in pre-implantation rhesus monkey embryos as well as male and female gametes studied using an optimized tagmentation-based whole-genome bisulfite sequencing method. We show that upon fertilization, both paternal and maternal genomes undergo active DNA demethylation, and genome-wide *de novo* DNA methylation is also initiated in the same period. By the 8-cell stage, remethylation becomes more pronounced than demethylation, resulting in an increase in global DNA methylation. Promoters of genes associated with oxidative phosphorylation are preferentially remethylated at the 8-cell stage, suggesting that this mode of energy metabolism may not be favored. Unlike in rodents, X chromosome inactivation is not observed during monkey pre-implantation development. Our study provides the first comprehensive illustration of the 'wax and wane' phases of DNA methylation dynamics. Most importantly, our DNA methyltransferase loss-of-function analysis indicates that DNA methylation influences early monkey embryogenesis.

## Introduction

DNA CpG methylation on the cytosine is among the most stable forms of epigenetic mechanisms in the life cycle of mammals. However, robust and large-scale genome-wide reprogramming of DNA methylome occurs during two critical developmental processes: (1) development of primordial germ cells and (2) pre-implantation embryogenesis. It is believed that such reprogramming primarily involves genome-wide active DNA demethylation, which apparently is crucial for re-setting the epigenetic states of the genome, allowing life cycle to restart and progress^1,2,3^.

Recently, genome-scale methylation sequencing of mouse and human gametes and pre-implantation embryos has been reported^4,5,6,7^. In mouse, a unidirectional demethylation process from the zygote stage to blastocyst stage is observed using either reduced representation bisulfite sequencing (RRBS) or single-base resolution whole-genome bisulfite sequencing (WGBS) method. In the human studies, the authors also observed a unidirectional demethylation during pre-implantation embryogenesis using RRBS^6,7^. Although the genome-wide DNA demethylation is believed to be a hallmark of mammalian embryogenesis, previous study also indicated that the somatic form of *dnmt1* (*dnmt1s*) is actually expressed at each stage of pre-implantation embryos and plays a role in the maintenance of DNA imprinting^8^. In 1-cell and 2-cell embryos Dnmt1s is derived from the oocyte, whereas from the 2-cell stage onward the embryo starts to synthesize its own Dnmt1s^8^. Other studies have also observed the expression of *de novo* DNA methyltransferases in early embryonic development^9,10^. These results strongly suggest the possibility of DNA remethylation during pre-implantation embryogenesis. However, probably due to technological limitations, no study has yet revealed genome-wide DNA remethylation during early embryogenesis. Furthermore, it is also of great interest to address how different DNA methylation dynamics is between primates and mouse, given that timing of zygotic genome activation and pre-implantation development is known to be divergent^11,12,13,14^. We therefore decided to investigate comprehensively the global and high-resolution DNA methylation dynamics during early development of a non-human primate (rhesus monkey, *Macaca mulatta*). To this end, we adopted a modified tagmentation-based whole-genome bisulfite sequencing (T-WGBS) method because, compared to all other genome-wide profiling strategies currently available, this method allows us to theoretically cover all CpG sites in the genome using small numbers of cells (100 cells)^15,16^.

Monkeys have served as one of the most valuable models for understanding DNA methylation dynamics during early embryogenesis in human due to their similarities in genetics and early embryonic development^17,18^. Furthermore, due to ethical and legal concerns, very limited techniques can be applied to human embryos to validate some of significant conclusions drawn from descriptive studies regarding human embryonic development. Therefore, studying methylome dynamics during early embryogenesis in a monkey model such as the rhesus monkeys is of great significance.

In this study, we performed comprehensive single-base resolution profiling of 5-methylcytosine (5mC) as well as gene expression analysis in monkey early embryos. In addition to demethylation, we observed *de novo* DNA methylation during pre-implantation embryogenesis, especially during the transition from the 2-cell to 8-cell stage. Most importantly, our loss-of-function experiments revealed that DNA methylation influences primate early embryogenesis. Our results refine the current knowledge on DNA methylation reprogramming in mammals and provide a valuable resource for future studies on primate embryonic development.

## Results

### Primate embryos display unique DNA methylation dynamics during pre-implantation development

To achieve genome-scale methylation profiling of primate pre-implantation embryogenesis with ultra-low input of DNA, we first modified a previously described transposase-based tagmentation bisulfite sequencing method^15,16^ (see Materials and Methods). The technical performance was first assessed on rice genome. Repeatability was confirmed by comparing replicates of libraries for CpG methylation with 0.5 ng total DNA yielding Pearson's correlation *R* of 0.97 (Supplementary information, Figure S1A, left), whereas accuracy was examined by comparing our T-WGBS with traditional chemistry ligation WGBS method in two experiments, resulting in Pearson's correlation *R* of 0.97 and 0.98 (Supplementary information, Figure S1A, middle and right). These results demonstrate the efficacy and accuracy of our optimized T-WGBS method for ultra-low input of DNA. We collected rhesus monkey sperm and ∼100 cells including MII-stage oocytes, zygotes, and cells from embryos at the 2-cell, 8-cell, morula stage, as well as cells from the inner cell mass (ICM) at the blastocyst stage (Supplementary information, Figure S2), to perform single-base resolution methylome sequencing using our optimized T-WGBS method. Highly reproducible data were generated from two samples of different embryos at each stage (Supplementary information, Figure S1B). On average, 60 41 840 CpGs at ×10 coverage were obtained for each sample (Supplementary information, Table S1). We used a highly stringent criterion, i.e., only cytosines that were covered for at least 10 times across all stages were included for subsequent analyses unless otherwise indicated.

**Figure 1:**
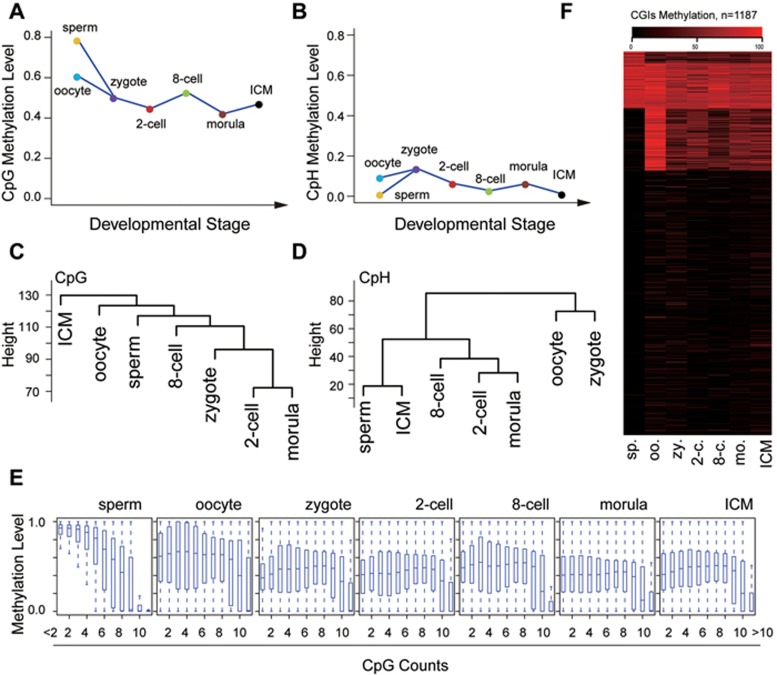
Global patterns of DNA methylome during monkey pre-implantation development. Averaged levels of DNA methylation at CpG **(A)** and CpH **(B)** sites at each stage of monkey early embryogenesis. Unsupervised hierarchical clustering of all filtered CpG **(C)** and CpH **(D)** sites in the genomes of sperm, oocytes, and embryos at different pre-implantation stages. Box plots show that with the exception of sperm, we do not observe an inverse relationship between CpG densities and methylation levels at other stages. CpG density was calculated as numbers of CpG sites in consecutive 100-bp tiles **(E)**. **(F)** Heatmap of methylation levels of CGIs across different developmental stages. 2-c., 2-cell stage; 8-c., 8-cell stage; ICM, inner cell mass at the blastocyst stage; mo., morula stage; oo., oocyte; sp., sperm; zy., zygote.

A Circos plot was first generated to display average CpG methylation levels within 500 kb windows across all 21 chromosomes (Supplementary information, Figure S3A). Apparently sperm has the highest average CpG methylation level (78.68%) across the whole genome, whereas oocytes have lower levels than sperm but still higher than the rest of the samples (Figure 1A and Supplementary information, Figure S3B). After fertilization, CpG methylation levels decrease rapidly in the zygotes (Figure 1A and Supplementary information, Figure S3B) due to active DNA demethylation, and reach the first lowest point at the 2-cell stage (44.8%). Interestingly, the average CpG methylation levels rise at the 8-cell stage, creating a small but significant peak at 52.7%. As development proceeds, the levels of DNA methylation decrease again, reaching the second lowest point at the morula stage (42.2%), which is followed by a gradual increase of DNA methylation at the blastocyst stage in the ICM cells (47.1%) (Figure 1A and Supplementary information, Figure S3B). This dynamic change was verified by immunostaining of 5mC (Supplementary information, Figure S4). Notably, as shown in Supplementary information, Figure S7A, re-analysis of human data^7^ reveals that remethylation also becomes more pronounced than demethylation in human embryos at the 8-cell stage, resulting in an increase in global DNA methylation levels, similar to what we observed in monkeys. Unbiased hierarchical clustering analyses demonstrate that DNA methylation patterns at the 2-cell and morula stages are most similar, probably because both genomes are highly hypomethylated (Figure 1C). No other two stages show tighter clustering, suggesting progressive changes in global CpG methylation patterns during early monkey embryonic development.

Unlike CpG methylation, the methylation levels of non-CpG (CpH) sites remain low and show much less change (Figure 1B and Supplementary information, Figure S3C). Nevertheless, demethylation and remethylation of CpH sites also occur (Figure 1B). The unsupervised clustering of methylation patterns at CpH sites resolves three clearly separated subgroups, different from the CpG patterns (Figure 1D). Although the functional consequences of non-CpG methylation are still poorly understood at present time, previous studies indicated that methylated non-CpGs are enriched in pluripotent stem cells, but rarely in somatic cells, suggesting a potential correlation between non-CpG methylation and pluripotency^19^. Therefore, our results on CpH methylation clustering might reflect different states of pluripotency during monkey early embryogenesis.

To determine whether CpG density might influence demethylation and remethylation preference, we plotted DNA methylation levels against CpG density levels across pre-implantation stages. Our data indicate that DNA methylation levels are high in low-density CpG sites in sperm, which is characteristic of somatic cells. However, for all the other stages, DNA methylation levels are not correlated with CpGs densities, except for the regions with high-density CpG sites, which mostly remain hypomethylated as in sperm (Figure 1E). High CpG density genomic regions are usually referred to as CpG islands (CGIs), most of which are predominantly non-methylated DNA sequences and function as regulatory elements in promoters by creating a transcriptionally permissive chromatin state^20^. Our k-mean clustering indicates most CGIs are unmethylated during the reprogramming process. However, sperm-specific and oocyte-specific hypermethylated CGIs are subject to reprogramming during development (Figure 1F). Furthermore, the hypomethylated CGIs are apparently not enriched for promoter regulatory regions (Supplementary information, Figure S3F).

Consistent with previous observations in mammals, the lowest CpG methylation levels are in the regions adjacent to the transcription start site (TSS), whereas exons, introns, and 3′ untranslated regions (3′ UTRs) are highly methylated (Supplementary information, Figure S5A). Such distribution is consistently found in genomes of both gametes and embryos across all examined pre-implantation stages, although sperm and oocytes generally have higher overall methylation levels than the early embryos (Supplementary information, Figure S5A and S5B). Different from other regions, CpG methylation levels in 5′ UTRs are hypomethylated, just like at CGIs, in gametes and embryos at all examined pre-implantation stages (Supplementary information, Figure S5B). We also examined DNA methylation dynamics of repeat elements, which is essential for mammalian pre-implantation development^21^, and found that the overall methylation dynamics of repeat elements including long-interspersed nuclear elements, short-interspersed nuclear elements, microsatellites, and etc., is similar to that of global DNA, except for rRNA regions, which remain hypomethylated (Supplementary information, Figure S5C).

### Active demethylation of paternal and maternal methylomes during primate pre-implantation embryogenesis

**Figure 2:**
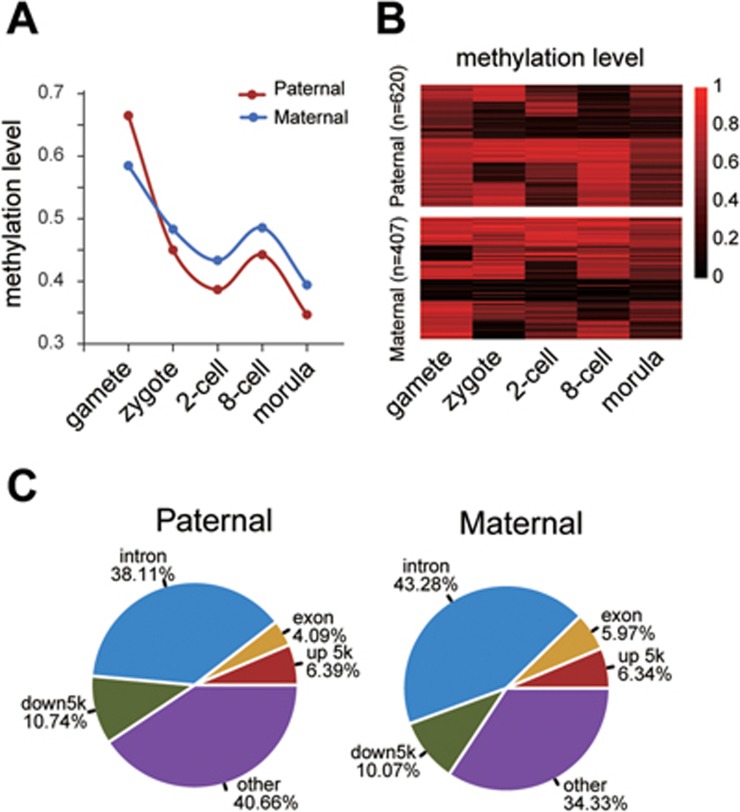
Monkey paternal and maternal DNA is actively demethylated. **(A)** Dynamics of CpG methylation reprogramming in paternal and maternal genomes during early embryogenesis. **(B)** Tracing of representative paternal-specific and maternal-specific CpG sites in oocytes, sperm, and other developmental stages. **(C)** Genomic distribution of paternal-specific and maternal-specific active demethylation CpG sites (relative demethylation level (RDL) value ≥ 0.6).

To address whether both paternal and maternal genomes similarly undergo active DNA demethylation or whether only one parental genome goes through this process, we applied Bis-SNP algorithm^22^ (see Materials and Methods) to call single-nucleotide polymorphisms (SNPs) to track paternal and maternal genomes. We obtained 5 070 SNPs whose parental origin could be clearly identified (Supplementary information, Table S2). Consistent with the global patterns, both the paternal and maternal DNA undergoes robust demethylation following fertilization, suggesting that active demethylation occurs on both paternal- and maternal-contributed DNA in the zygotes. From the 2-cell stage and onward, the paternal and maternal genomes display identical patterns of DNA methylation dynamics (Figure 2A). When we tracked methylation changes of 2 058 paternal and 1 173 maternal CpG sites during the transition from gametes to zygotes, we observed both demethylation and remethylation occurring in a significant portion of paternal and maternal CpG sites. There are also large portions of CpG sites that maintain their methylation levels (Figure 2B). These data clearly indicate only a portion of paternal and maternal DNA undergoes active demethylation right after fertilization as no cell division takes place during this period. When the distribution of active demethylation CpG sites with the relative demethylation level value ≥ 0.6^5^ is plotted, both paternal and maternal actively demethylated CpG sites in zygotes are located mainly in intronic and intergenic genomic regions (Figure 2C).

### Pairwise comparisons revealing *de novo* DNA methylation accompanying demethylation during pre-implantation embryogenesis

**Figure 3:**
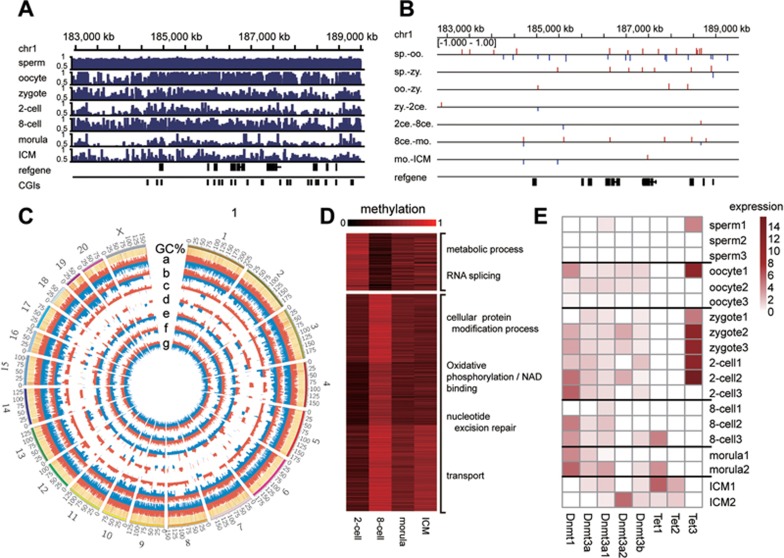
Developmental transitions in CpG methylation revealed by pairwise comparisons. **(A)** Graphical representation of a genomic region on chromosome 1 showing methylation levels of CpGs in gametes and at different developmental stages before implantation. **(B)** DMR distribution in a representative genomic region on chromosome 1. Red lines indicate DMRs that are previously hypermethylated and demethylated during embryogenesis; blue lines indicate DMRs that are previously hypomethylated and remethylated during embryogenesis. **(C)** Circos plot of the genome-wide distribution of DMRs in several pairwise comparisons. a, sperm vs oocytes; b, sperm vs zygotes; c, oocytes vs zygotes; d, zygotes vs 2-cell stage embryos; e, 2-cell stage embryos vs 8-cell stage embryos; f, 8-cell stage embryos vs morula stage embryos, and g, morula stage embryos vs ICM from the blastocyst stage. Red lines indicate DMRs that are previously hypermethylated and demethylated during embryogenesis; blue lines indicate DMRs that are previously hypomethylated and remethylated during embryogenesis. **(D)** GO analysis of DMRs between the 2-cell and 8-cell stages indicating that oxidative phosphorylation and nucleotide excision repair pathways may be silenced or repressed at the 8-cell stage (metabolic process, *P* = 0.021; RNA splicing, *P* = 0.026; cellular protein modification process, *P* = 0.007; oxidative phosphorylation, *P* = 0.034; nucleotide excision repair, *P* = 0.028; transport, *P* = 0.049). **(E)** log_2_-normalized qPCR expression of *dnmts* and *tet1/2/3* at different stages. 2ce., 2-cell stage; 8ce., 8-cell stage; ICM, inner cell mass at the blastocyst stage; mo., morula stage; oo., oocyte; sp., sperm; zy., zygote.

The unexpected rise of DNA methylation at the 8-cell stage suggests the presence of *de novo* DNA methylation during pre-implantation embryogenesis. To explore further we first focused on a randomly selected representative region on chromosome 1. In this region, as shown in Figure 3A, sperm DNA is highly methylated, whereas oocyte DNA is less methylated albeit it is more than zygote DNA. Pairwise comparisons between sperm and oocytes (Sp-Oo) demonstrate that sperm-specific high CpG methylation regions and oocyte-specific high methylation regions are intermingled (Figure 3B). Similar pattern is also found when sperm DNA is compared with zygote DNA (Sp-Zy). The zygote-specific high methylation levels may mainly come from not yet demethylated maternal DNA, or some of the low-level *de novo* DNA methylation at the zygote stage. Similarly, when oocyte DNA methylation levels are compared to those of zygotes (Oo-Zy), oocyte-specific high methylation regions (red lines in Figure 3B) are likely demethylated in zygotes after fertilization, whereas zygote-specific high methylation regions (blue lines in Figure 3B) likely gain methylation from either not-yet demethylated sperm DNA, or some levels of *de novo* DNA methylation in the zygotes. As development proceeds, both demethylation (active and passive) and *de novo* methylation occur, with overall remethylation dominating over demethylation at the 8-cell stage, and demethylation dominating remethylation after the 8-cell stage and onto the morula stage. At the blastocyst stage *de novo* DNA methylation gains additional momentum, causing a gradual rise of the global DNA methylation. Pairwise comparisons at a genome-wide scale demonstrate that the aforementioned trend of DNA methylation dynamics is not specific for chromosome 1, but rather, occurs throughout the whole genome (Figure 3C and Supplementary information, Table S3). Notably, de novo DNA remethylation also occurs during human pre-implantation development (Supplementary information, Figure S7B-S7D). In monkey, differentially methylated regions (DMRs) are located in both gene body and other intergenic regions (Supplementary information, Figure S6A). Apparently, the 8-cell stage is a key turning point for major waves of remethylation. We found that promoters that gain methylation at this point reside in genes that are associated with oxidative phosphorylation (gene ontology, or GO, terms with *P*-value < 0.05). As hypermethylation of promoters may hamper gene transcription initiation^23^, this result suggests that this form of energy metabolism might not be favored during early embryogenesis; instead, anaerobic energy metabolism could be in use^24^ (Figure 3D). Interestingly, most of these remethylated promoters are subject to demethylation at the later stage of monkey embryogenesis (Figure 3D). Moreover, the majority of genes gaining methylation in the promoter regions at the 8-cell stage in human embryos are also related to metabolic processes such as glucose metabolism regulation (Supplementary information, Figure S7E and Supplementary information, Table S4).

Gene expression data demonstrate that major demethylase genes (*tet 1,2,3*)^25^ are expressed throughout the pre-implantation period, with *tet3* being expressed early during the major waves of active demethylation and *tet1/2* being expressed from the 8-cell stage onward (Figure 3E). At the 8-cell stage *tet3* expression is eliminated while tet1/2 expression is just about to begin, presumably rendering the lowest demethylation activity to cells. On the other hand, genes encoding DNA methyltransferases (*dnmts*) are also expressed throughout the pre-implantation period, which supposedly mediate the global DNA remethylation during this period.

The above results support our hypothesis that pre-implantation developmental dynamics of DNA methylation is not unidirectional in primate; instead, a dynamic equilibrium between demethylation and remethylation exists, which is presumably subject to multifaceted complex regulation.

### Characteristics of paternal or maternal hypermethylated CpG sites and X chromosome inactivation-related features during pre-implantation embryogenesis

**Figure 4:**
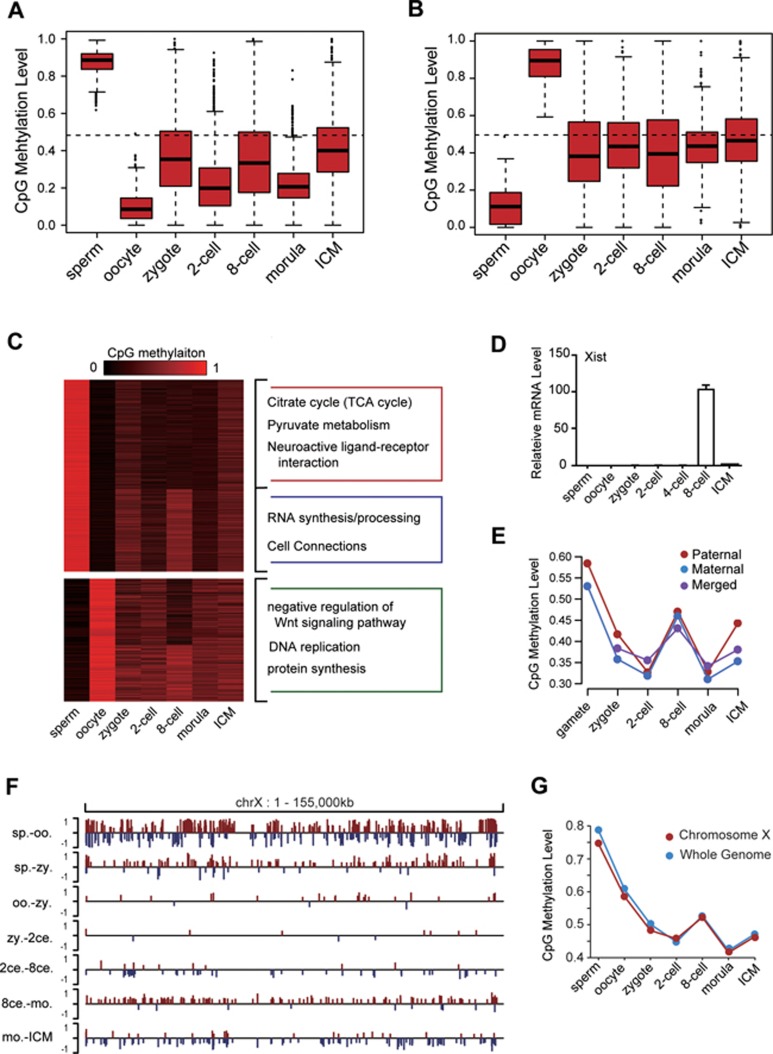
Tracing of gamete-specific hypermethylated CpG sites and X chromosome inactivation during early embryogenesis. Box plot of methylation levels at sperm-specific hypermethylated sites (methylation level > 0.75 in sperm and < 0.25 in oocytes, *n* = 2365) **(A)** and at oocyte-specific hypermethylated sites (methylation level > 0.75 in oocytes and < 0.25 in sperm, *n* = 5 356) **(B)** during early pre-implantation development. The average methylation level is indicated by the dashed line. **(C)** Heatmap of DNA methylation dynamics in sperm-specific hypermethylated promoters and oocyte-specific hypermethylated promoters during pre-implantation embryogenesis. GO analysis indicates that genes with oocyte-specific hypomethylated promoters are enriched in the citrate cycle pathway, the pyruvate metabolism pathway, and cell connections, whereas genes with sperm-specific hypomethylated promoters are enriched in negative regulation of the Wnt signaling pathway and DNA replication (GO terms with *P*-value < 0.05). **(D)** RT-qPCR analysis of *xist* expression during early embryogenesis relative to the expression of *gapdh* (encoding glyceraldehyde phosphate dehydrogenase) (*n* = 3 per stage). **(E)** Methylation levels of maternal-specific and paternal-specific X chromosome CpG sites during early embryogenesis. **(F)** Graphical representation of DMR distribution in a representative genomic region of X chromosome. **(G)** Dynamics of the methylation level on X chromosome follows a similar pattern to the global methylation.

Due to the significant differences between the sperm and oocyte methylomes, we tracked sperm-specific hypermethylated CpGs (CpG methylation levels > 75% in sperm and < 25% in oocytes) and oocyte-specific hypermethylated CpGs (CpG methylation levels > 75% in oocytes and < 25% in sperm) at various stages of pre-implantation development. We found that methylation dynamics of sperm-specific sites closely resembles the global DNA methylation patterns, whereas oocyte-specific hypermethylated sites, once mixed/diluted by sperm DNA upon fertilization, do not change significantly (Figure 4A and 4B). These maternal sites might be maternally imprinted. GO analysis reveals that genes with hypermethylated promoters in sperm are related to citrate cycle and pyruvate metabolism, whereas hypermethylated genes in oocytes are related to DNA replication, protein synthesis, and negative regulation of the Wnt signaling pathway (Figure 4C) (GO terms with *P*-value < 0.05).

Surprisingly, a gene involved in X chromosome inactivation (XCI), *xist*, is transiently expressed in 8-cell stage female embryos, when genome-wide *de novo* DNA methylation exceeds global DNA demethylation (Figure 4D). Remethylation also occurs on X chromosomes from both parents during pre-implantation development (Figure 4E). However, this remethylation is again erased at the morula stage, suggesting the transient X chromosome remethylation is not stable and may participate in XCI (Figure 4E). Moreover, when CpG sites within the maternal- and paternal-specific SNPs on X chromosomes were surveyed, no XCI-specific methylation patterns were observed, suggesting that XCI does not occur during monkey pre-implantation embryogenesis (Supplementary information, Figure S8). Finally, the overall DNA methylation dynamics of the X chromosomes is almost identical to that of the whole genome, again suggesting no X chromosome-specific mode of DNA methylation regulation occurs throughout monkey pre-implantation development (Figure 4F and 4G). Although xist expression pattern during human early embryogenesis is identical to that of monkey^26^, it is unclear if there exists a relationship between xist expression and X chromosome methylation levels. Moreover, it remains to be determined whether xist expression at the 8-cell stage is functional, and if it is, what that function would be.

### Cross-comparisons of monkey and mouse methylome dynamics during early embryonic development

**Figure 5:**
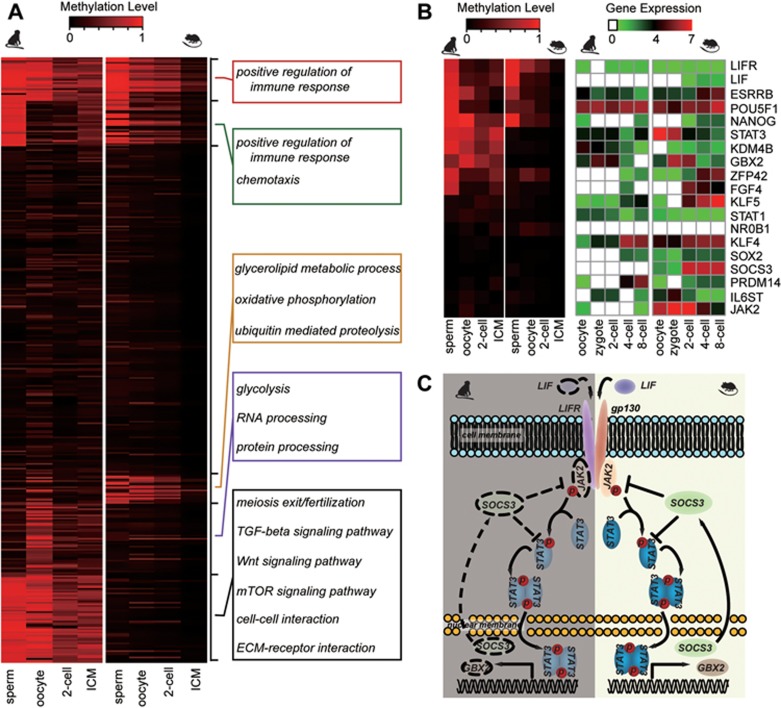
DNA methylation dynamics during pre-implantation embryogenesis is different between monkey and mouse. **(A)** Heatmap of promoter DNA methylation levels of orthologous genes during monkey and mouse pre-implantation development. Monkey genes are mapped to orthologous mouse genes from the Mouse Genome Informatics (MGI) database. **(B)** Gene expression and methylation levels in the promoter region of naive genes in monkey and mouse. **(C)** The LIF/STAT3 signaling pathway is inactivated in monkey.

Comparison of DNA methylation dynamics in mouse^5^ and monkey embryonic development side by side by k-means clustering reveals that the methylation dynamics of monkey is significantly different from that of mouse (Figure 5A). At the blastocysts stage, most orthologous loci are hypomethylated in mouse, but remain hypermethylated in monkey. Interestingly, the pluripotency maintenance-related TGFβ and Wnt signaling pathways are differentially methylated in mouse and monkey (Figure 5A). We investigated acquisition of pluripotency between mouse and monkey within the same pre-implantation timeframe. In accordance with promoter methylation levels, many known naive-state genes, such as *esrrb*, *zfp42*, *fgf4*, and *gbx2*, are expressed at lower levels in monkey than in mouse, especially in ICM cells. Interestingly, members of the LIF/STAT3 signaling pathway, which plays an important role in maintaining naive state of mouse embryonic stem cells, are differentially regulated in monkey and mouse both at the DNA methylation and RNA expression levels. We found methylation levels in the promoters of *lif*, *socs3*, and *jak2* are high, and their expression is more suppressed, in monkey (Figure 5B and 5C). Our data provide evidence that naive state acquisition is regulated differently in primate and mouse.

### Knockdown of dnmt3a and dnmt3b at zygote stage causes more *in vitro* fertilization embryos to reach the blastocyst stage

**Figure 6:**
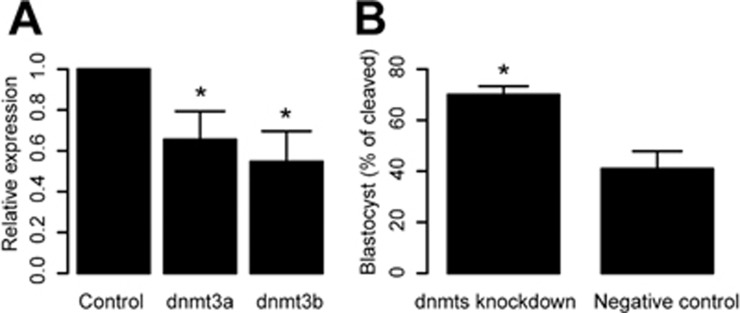
*dnmt3a* and *dnmt3b* knockdown improves developmental potential of the embryos. **(A)** Morpholinos can significantly knock down endogenous *dnmt3a* and *dnmt3b* expression in monkey embryos (*n* = 3, ^*^ represents *P* ≤ 0.05). **(B)** Bar graph showing percentages of embryos that reach the blastocyst stage after injection of *dnmt3a* and *dnmt3b* morpholinos or negative control morpholino (Three independent experiments. Totally 15 embryos were used in control and knockdown experiments, respectively. Error bars represent SD. ^*^ represents *P* ≤ 0.05).

During *in vitro* fertilization (IVF) of rhesus monkey, developmental failure often occurs at the transition from the 8-cell stage to the morula stage. As accurate epigenetic reprogramming is essential in normal development, the failure may result from incomplete demethylation or remethylation caused by imperfect *in vitro* culture condition. On the basis of the observation we described above, we hypothesized that blockade of *de novo* DNA methylation during the pre-implantation period could influence embryonic development. To test this hypothesis we injected morpholinos against *dnmt3a* and *dnmt3b* at the zygote stage; and found that the number of embryos arrested at the transition from the 8-cell to the morula stage was indeed significantly reduced: 70% of embryos injected with *dnmt3a* and *dnmt3b* morpholinos developed into blastocysts, whereas only about 40% of the negative control embryos developed into the blastocyst stage, which is expected from IVF in monkey (Figure 6A and 6B).

Taken together, these observations suggest that DNA remethylation negatively regulates pre-implantation development of monkey embryos derived from IVF. It is unclear whether *de novo* DNA methylation during pre-implantation development also occurs in normal embryos. If it is, this early remethylation process may help set a stage for the development after the blastocyst stage, when *de novo* DNA methylation becomes crucial. If the *de novo* DNA methylation in the pre-implantation phase is unique to IVF embryos, it implies that the culture conditions currently used in IVF procedure are not optimized, which can induce abnormal *de novo* DNA methylation, and inhibit early embryonic development.

## Discussion

Using unbiased genome-wide bisulfite sequencing with ultra-low DNA input, we performed high-resolution, high-coverage methylome profiling of rhesus monkey pre-implantation embryos. Unlike those reports using mouse pre-implantation embryos with classic WGBS, which demonstrate that DNA methylation dynamics is a stable and mostly unidirectional DNA demethylation process, our data indicate that in early monkey embryos, DNA methylation dynamics exhibits a 'wax' and 'wane' pattern, with both DNA demethylation and *de novo* remethylation taking place during development, producing a DNA methylation peak at the 8-cell stage preceded and followed by phases of low methylation at the 2-cell stage and the morula stage. It is conceivable that the remethylation adds an additional dimension to the regulation, fine-tuning the gene expression to mediate complex development processes. Recently, remethylation was observed in the zygote stage in mouse^27^, suggesting DNA remethylation is a conserved regulatory feature in mammalian development. Moreover, the increased methylation of genes associated with oxidative phosphorylation may indicate a bias toward using anaerobic energy metabolism during this period of development, which is consistent with the metabolic features of stem cells recently described^24^. Moreover the majority of genes that gain methylation at their promoter regions at the 8-cell stage of human embryogenesis are also related to metabolic processes. This suggests that regulation of metabolism at the 8-cell stage is a critical event during primate pre-implantation development.

Monkeys are one of the most valuable models for modeling human diseases and studying human early embryogenesis^17,18^. Our reanalysis of the data in published human studies^7^ also reveals traces of *de novo* DNA methylation across all stages of early human embryogenesis. This suggests that DNA remethylation is a common phenomenon during early primate development. The highly complex regulation of DNA methylation dynamics during the pre-implantation phase of primate embryogenesis may be very important, yet labile to embryonic manipulations *in vitro*, which can contribute to the low success rate of animal cloning via somatic cell nuclear transfer in primates as compared to rodents.

Similar to what was found in mouse zygotes, DNA demethylation of the maternal genome is not a cell cycle-dependent, passive demethylation process, but rather a *tet-3*-mediated active DNA demethylation event, exactly the same as the DNA methylation process of the paternal genome^28,29^. When paternal alleles and maternal genome were studied separately using SNPs, we found that after the 2-cell stage, the overall DNA methylation dynamics is very similar between the paternal alleles and maternal alleles. Disruption of the expression of *dnmt3a* and *dnmt3b* immediately after fertilization significantly promoted the development of monkey embryos. We speculate that during early pre-implantation development, DNA methylation reprogramming is critically important. To develop a comprehensive perspective of remethylation process during monkey early embryonic development, we plotted all identified DMRs across 21 chromosomes. Interestingly, remethylation takes place as early as the zygote stage, and throughout the entire early embryonic development in monkey. As remethylation has not been reported in rodent and has not been carefully investigated in human, it will be interesting to determine whether this is a general phenomenon in mammals or only occurs in primates, or whether it occurs both *in vivo* and *in vitro* after IVF. Moreover, the biological function of *de novo* DNA remethylation during early embryonic development remains to be further defined in the future.

## Materials and Methods

### Collection of monkey sperm, oocytes, and early embryos

Rhesus monkey (*M. mulatta*) ovarian stimulation and oocyte recovery were performed as previously described^18^. Briefly, adult females were subject to follicular stimulation using twice daily intramuscular injection of 18 IU of recombinant human FSH (rhFSH, Gonal F, Laboratories Serono SA) for 8 days; then 1 000 IU of human chorionic gonadotropin (rhCG, OVIDREL, Merck Serono) was injected on day 9. Cumulus-oocyte complexes were collected from animals by laparoscopic follicular aspiration 32-35 h following rhCG administration. Follicular contents were placed in Hepes-buffered Tyrode's albumin lactate pyruvate (TALP) medium containing 0.3% BSA at 37 °C. Cumulus-oocyte complexes were exposed to hyaluronidase (0.5 mg/ml, < 1 min) in TALP-Hepes to strip off cumulus cells. Oocytes that were mature (MII) at collection were placed in chemically defined, protein-free hamster embryo culture medium-10 (HECM-10) at 37 °C in 5% CO_2_ until they were inseminated (within 24 h) with capacitated, hyperactivated spermatozoa diluted to a final concentration of 2 × 10^5^/ml in 50-μl drops of TALP for fertilization. After co-incubation of oocytes and spermatozoa for 12-16 h, oocytes were examined for the presence of two pronuclei and two polar bodies as evidence of fertilization. Then the polar bodies were mechanically dissected using Piezo-Micromanipulator to eliminate the DNA contamination of polar bodies and fertilized oocytes were washed to remove sperm and then cultured in HECM-10 containing 10% FCS (Hyclone) to allow embryo development. Culture medium was replaced every 2 days. The embryos were collected at different stages (zygote; two-pronuclear (2 PN) stage, 14-16 h post fertilization (hpf); 2-cell stage, 24-30 hpf; 8-cell stage, 2 days post fertilization (dpf); morula, 4-5 dpf; blastocyst, 6-7 dpf). Zonae pellucidae of embryos were removed by brief exposure (45-60 s) to 0.5% pronase in culture medium. For ICMs collection, zona-free blastocysts were exposed to rabbit anti-rhesus spleen serum (Axell Labs) (1:5) for 30 min at 37 °C. After extensive washing in ESC derivation medium, embryos were incubated in guinea pig complement (1:5) for an additional 30 min at 37 °C to facilitate the removal of trophectoderm cells from ICMs by pipetting through a fine-pulled glass needle. All samples were carefully washed in PBS-EDTA to remove any contaminants. All chemicals were from Sigma Chemicals unless otherwise stated.

Total 25 oocyte donor monkeys were used in this study and with the exception of ICM (from brother German), all the sperm was derived from the same male monkey. All animals were housed at the Kunming Biomed International, an Association for Assessment and Accreditation of Laboratory Animal Care-accredited facility. All animal protocols were approved in advance by the Institutional Animal Care and Use Committee of Kunming Biomed International.

### T-WGBS library construction and sequencing

Transposome complex was first generated by incubating 2.5 μl of 10 μM annealed adaptors with 2.5 μl 100% glycerol and 5 μl Ez-Tn5 transposase (Epicentre, Illumina) for 30 min at 25 °C. Cells were lysed by proteinase K treatment for 40 min at 37 °C. The genomic DNA was purified by AMPure XP magnetic beads (Beckman Coulter). The purified DNA (∼ 0.5 ng, spiked with 5 pg of unmethylated lambda DNA) was then incubated with 4 μl Nextera HMW Buffer (Epicentre-Illumina), 16 μl nuclease-free water (Ambion), and 4 μl prepared Tn5mC transposome complex for 12 min at 55 °C followed by purification using 36 μl (1.8×) Agencourt AMPure XP magnetic beads; and the DNA was eluted in 14 μl EB buffer (Qiagen). An extension step was then performed by adding 2 μl of 10× Thermopol reaction buffer (New England Biolabs), 2 μl 10 mM 5mC dNTP Mix (Zymo Research), 1 μl of Bst DNA polymerase large fragment (New England Biolabs) to each reaction mixture and incubated for 20 min at 65 °C. Each reaction was spiked with 200 ng of sonicated unmethylated lambda DNA (200-400 bp) (Takara) and then subject to bisulfite conversion using a Zymo EZ DNA Methylation Kit (Zymo Research) following manufacturer's protocols involving a 14 h 50 °C incubation at dark and 22 μl (2 × 11 μl) elution. The purified DNA was then amplified using 25 μl Kapa 2G robust hot start ready mix (Kapa Biosystems), 1 μl 50× Nextera primer cocktail (Illumina – compatible) and 1 μl barcoded Illumina-compatible adaptor 2 (8-bp index) on a thermocycler with the following parameters: 1 min at 95 °C followed by 10-15 cycles of 20 s at 95 °C, 30 s at 60 °C, 45 s at 72 °C. The prepared libraries were analyzed by Agilent 2100 Bioanalyzer (Agilent Technologies) and quantified by Quantitative PCR (qPCR) and then used for cluster generation and pair-end sequencing with 90 bp reads (PE90) on Illumina Hiseq 2000 (Illumina).

### Chemistry ligation-based WGBS library construction and sequencing

Chemistry ligation-based WGBS libraries were generated from genomic DNA (each library was spiked with 1 ng of unmethylated lambda DNA) based on the methods described previously^23^. According to the standard Illumina protocol, libraries were sequenced using a strategy with pair-end 90 bp read (PE90) on Illumina Hiseq 2000 (Illumina).

### Read filtering and mapping

After sequencing, adapters were removed using cutadapt-1.2.1 software (http://code.google.com/p/cutadapt/), only reads with minimally 45 bp length were kept. After removing the adapters, low-quality reads that contained more than 10% Ns or more than 50% of the sequence with low-quality value (quality value < 5) per read were filtered out. Reference genome sequences were downloaded from UCSC genome browser (rheMac3); only the assembled chromosomes were used. Reads mapping was done using BS-MAP (version 2.73) alignment software^30^ that combines genome hashing and bitwise masking to achieve fast and accurate bisulfite mapping, allowing for maximally five mismatches or one small insertion or deletion. After mapping, PCR duplicates were removed using Samtools software^30^. Then, we separated the reads into two categories, depending on whether they are from the top (C changed to T, C-T) and bottom (G changed to A, G-A) strands.

To re-analyze the human RRBS bisulfite sequencing data, the single CpG methylation levels were downloaded from the NCBI Gene Expression Omnibus (GEO) under accession number GSE49828.

### Quantification of methylation levels

Methylation levels are defined as the fraction of read counts of 'C' in the total read counts of both 'C' and 'T' for each covered C site, and herein average percentage methylation of all cytosine residues for any genomic region covered was computed as the fraction of read counts of 'C' in the total read counts of both 'C' and 'T' for each genomic region. On the basis of such read fraction, methylated cytosine was called using a binomial distribution as in the method described by Lister *et al*.^23^, whereby a probability mass function is calculated for each methylation context (CpG, CHG, CHH). The non-conversion rate and sequencing error rate were taken into account, for which the total error rate was determined by unmethylated lambda DNA spike-ins.

For human, the methylation level of each sampled cytosine was estimated as the number of reads reporting a C, divided by the total number of reads reporting a C or T. Only the CpG sites with read coverage more than five times were taken into consideration.

### Identification of pairwise differentially methylated regions

DMRs were searched using a sliding window strategy: first, we selected CpG sites covered in two samples with sequencing depth ≥ 5× as candidate sites. For each of candidate site, the ChiqX or Fisher test was performed to calculate the significant test *P*-value. Second, we selected the first differentially methylated CpG (*P*-value < 0.05) as an initial locus of DMR, and began to merge these candidate sites into a candidate DMR with following criteria: (1) the distance between two neighboring candidate sites was ≤ 300 bp; (2) all of candidate sites in the candidate DMR kept the same methylation status (either hypermethylated or hypomethylated); (3) a candidate DMR must harbor 10 or more candidate sites. For each of above candidate DMRs, we performed a Fisher test again, and filtered out those regions whose test *P*-value was > 0.01 and the difference of mean methylation levels between two samples was < 0.3.

### Annotation of gene and genomic features

Gene annotations were downloaded from UCSC genome browser (rheMac3)'s Refseq and Genscan tracks. For Genscan gene annotation, BLAST was applied to further annotate the gene functions because the current annotation is not complete. All the predicted coding DNA sequence (CDS) regions of Genscan genes were aligned using BLAST with human and monkey sequences in the database. A cutoff E-value < 1e−05 was applied in filtering, and a best alignment term for each query CDS sequence was selected if more than one query sequence was aligned based on this cutoff E-value from BLAST. Promoters were defined as 2 kb upstream and 1 kb downstream of the TSS. Repeat sequence annotations were downloaded from the UCSC browser (rheMac3)'s RepeatMasker tracks. CGIs were defined as regions > 200 bp with a GC fraction > 0.5 and an observed-to-expected ratio of CpG > 0.65 as annotated in UCSC genome browser. Mouse gene annotation data were also obtained from the UCSC genome browser (mm10); corresponding human gene annotations were downloaded from the UCSC genome browser (hg 19).

### GO annotation and pathway enrichment analysis

GO analysis of genes containing DMRs near the TSS was performed using DAVID (http://david.abcc.ncifcrf.gov/) and Gostat (http://gostat.wehi.edu.au/cgi-bin/goStat.pl). GO terms with *P*-value < 0.05 were considered to be statistically significant. WebGestalt (http://bioinfo.vanderbilt.edu/webgestalt/option.php) was used for Kyoto Encyclopedia of Genes and Genomes pathway enrichment analysis. Pathways with adj *P*-value < 0.05 were determined to be statistically significant pathways.

### Identification of SNPs and parent-of-origin methylation tracking

Bis-SNP tool^22^ which can identify mono-allelic DNA methylation and polymorphisms in *cis*-regulatory sequences was used to detect SNPs in our DNA bisulfite treatment sequencing data. First, 3 25 327 SNPs were identified in zygotes according the following filtering criteria: (1) a SNP had to be covered by at least 10 reads; (2) the minimum base quality for calling a base was 5; (3) the minimum phred-scaled confidence threshold at each variant for calling should be 20; (4) the heterozygous SNP genotype quality score should be > 20. Second, each heterozygous SNP in zygotes with both alleles depth ≥ 10× was used to retrieve the genotype in other stages, and SNPs which had consistent genotype in the same position were kept. Third, if a highly confident heterozygous SNP contained an allele that was the same as the sperm genotype, this SNP was used to trace the maternal and paternal origin of the reads. After these steps, 5 070 SNPs were obtained and used to track allele-specific DNA methylation. Maternally derived reads and paternally derived reads were distinguished according to the SNP, and CpG methylation levels were called in the same manner described above.

### Preparation of single-cell cDNA

Preparation of single-cell cDNA was performed using previously published protocols^31,32^. Briefly, single cells were randomly picked from monkey embryos under microscope and lysed in a cell lysis buffer. mRNA in the lysate was reverse transcribed into cDNA by SuperScript III reverse transcriptase (Invitrogen) with a poly (T) primer having an anchor sequence (UP1) at its 5′ end in a 60 min reaction at 50 °C. Subsequently, the reverse transcriptase was inactivated at 70 °C for 15 min. To increase the reverse transcription efficiency, dNTP concentration was raised from 0.225 to 0.5 mM in the reverse transcription step and ExoSAP (USB Corporation) was used to remove free dNTP and primer after the reaction. A poly (A) tail was added to the first-strand cDNA at the 3′ end by terminal deoxynucleotidyl transferase (Life Technologies). Following the second-strand cDNA synthesis with TaKaRa Ex Taq HS DNA polymerase (Takara) and a poly (T) primer containing a different anchor sequence (UP2) at the 5′ end, the cDNA was amplified using the following condition: 18 cycles of 95 °C for 30 s, 67 °C for 1 min, 72 °C for 8 min with an extra 6 s added in each cycle. The amplified cDNA was purified with QIAquick PCR purification Kit (Qiagen) and was further amplified for 9 cycles using poly (T) primers with an anchor sequence containing a 5′ end blocked by amine at the C6 position (NH2-UP1 and NH2-UP2). Afterward, the amplified cDNA with sizes ranging from 0.5 to 8 Kb was excised on a 1% agarose gel; Agilent 2100 Bioanalyzer and real-time TaqMan assay were used to determine the yield and quality of the final cDNA samples. These cDNA libraries, which included the majority of expressed genes were sequenced or analyzed by quantitative real-time PCR analysis.

### Quantitative real-time PCR

qPCR analysis was performed according to the manufacturer's instructions and monitored with SYBR green PCR master mix (SYBR Premix Ex Taq II, Takara) using the MyiQ2 Real-Time PCR Detection System (Bio-Rad) to check the expression of housekeeping gene *gapdh* and specific genes *dnmt1*, *dnmt3a*, *dnmt3a1*, *dnmt3a2*, *dnmt3b*, *tet1*, *tet2*, and *tet3*. All reactions were conducted in triplicate.

The following primers were used:

*dnmt1*: sense 5′-GGGAAGTGAATGGACGTCTAGAAA-3′,

antisense 5′-TCTGGTGCTTTTCCTTGTAATCCT-3′

*dnmt3a*: sense 5′-GCTGAGCTCGTTTTGCAGTGCGTT-3′,

antisense 5′-ACCTCCACGGCCTTGGCAGTGTCAC-3′

*dnmt3a1*: sense 5′-GAACAGAAGGAGACCAACATCGAA-3′,

antisense 5′-GCGCTTGCTGATGTAGTAGGG-3′

*dnmt3a2*: sense 5′-ATTCAGGTGGACCGCTACATTGCC-3′,

antisense 5′-GGATATGCTTCTGTGTGACGCTGC-3′

*dnmt3b*: sense 5′-TGGGGCATACCGTGTACCTCAGTT-3′,

antisense 5′-TTTTAGGAGAAGAAAAAATGAGCAC-3′

*tet1*: sense 5′-TCTGTAGCTATGTCTCGATCCCGC-3′,

antisense 5′-ATTTTTTTTGTTGGCTCCCTTGGGTG-3′

*tet2*: sense 5′-CGTAGAGAAGCAGAAGGAAGCAAG-3′,

antisense 5′-CAGGAGCAAAGGCAAGTAAACAAT-3′

*tet3*: sense 5′-CTGGGGTTGATGGATGAATGGTAG-3′,

antisense 5′-GAGGGAGAAGGGAGGGTAAAGGAG-3′

*gapdh*: sense 5′-CCCATCACCATCTTCCAGGAGCGA-3′,

antisense 5′-ATGATGACCCTTTTGGCTCCCCCC-3′

### dnmt3a and dnmt3b morpholinos design and embryo injection

We used two morpholinos to knockdown *dnmt3a* and *dnmt3b* protein synthesis in monkey embryos (www.gene-tools.com). The sequences of morpholinos against *dnmt3a* and *dnmt3b* are 5′-GGTTTTCTTCCACAGCATTCATTCC-3′ and 5′-ATGCCTGGTGTCTCCCTTCATGCTT-3′, respectively. The sequence of negative control morpholino, which does not target any gene in monkey, is 5′-ATCCCTGCTGTCTACCTTAATGATT-3′. For injections, morphplinos of *dnmt3a*, *dnmt3b*, and negative control morpholino were dissolved in culture-grade H_2_O at a concentration of 1 mM. Equal amounts of *dnmt3a* and *dnmt3b* morpholinos were mixed together and injected into zygote stage embryos. The injections were done in triplicate, and totally 30 embryos were injected, of which 15 were used for *dnmts* morpholinos injection and other 15 were used for negative control morpholino injection.

### Immunostaining of the early embryos

Early monkey embryos were collected and washed with PBS thoroughly. The samples were fixed with 4% paraformaldehyde in PBS for 15 min and permeabilized with 0.5% Triton X-100 in PBS for 1 h. After three washes in 0.05% Tween-20 in PBS, all samples were treated with 4 M HCl for 10 min and fully rinsed again with 0.05% Tween-20 in PBS. After washing, samples were blocked in blocking solution (1% BSA and 0.05% Tween-20 in PBS) overnight at 4 °C. The embryos were next incubated with anti-5mC antibody (1:100, Abcam) at 25 °C for 1 h, washed three times, and incubated with Alexa Fluor 488 goat anti-mouse IgG (1:500, Invitrogen) for 1 h at 25 °C. DNA was labeled with propidium iodide at a concentration of 10 μg/ml. Isotype-matched IgG was used as the negative control in each experiment.

### Data access

All sequencing data were deposited at the NCBI GEO under accession number GSE60166. Human pre-implantation embryos RRBS sequencing data can be obtained from GEO under Accession Number GSE49828.

## Author Contributions

FG, TT, WJ designed the study. FG, YS, HL, SL, JY, and TT analyzed the data and performed statistical analyses. YN, YC, YK, CS, and TT performed samples collection work including superovulation, micromanipulation, animal care, etc. FG, SL, TT, YL, CL, and NS carried out experiments or contributed critical reagents and protocols. WS and HW assisted in monkey work. FG and HL were involved in sequencing. TT, YS, FG, HL, and WJ wrote the manuscript.

## Competing Financial Interests

The authors declare no competing financial interests.

## Supplementary Material

Supplementary information, Figure S1 The sensitivity and reproducibility of T-WGBS technique.

Supplementary information, Figure S2 Isolation of monkey embryonic samples for T-WGBS.

Supplementary information, Figure S3 Global patterns of DNA methylation during monkey early embryogenesis.

Supplementary information, Figure S4 DNA methylation patterns in monkey embryos detectedby immunostaining.

Supplementary information, Figure S5 DNA methylation dynamics in different genomic elements.

Supplementary information, Figure S6 Sequential pairwise comparisons between gametes and all pre-implantation stages.

Supplementary information, Figure S7 *de novo* DNA methylation across all stages of early human embryogenesis.

Supplementary information, Figure S8 Methylation dynamics of paternal and maternal specific loci in X chromosomeduring early embryogenesis.

Supplementary information, Table S1 Summary of the sequencing qualities, the covered CpG sites and their meancoverage depths at 1×, 5× in each embryonic stage.

Supplementary information, Table S2 The list of called SNPs positions, SNP genotypes, and the chromosome locations.

Supplementary information, Table S3 The list of all pairwise comparisons derived DMR regions.

Supplementary information, Table S4 Functional classification of biological process of genes with hypermethylated promoters at human 8-cell stage.
