# Supplementary material for: De novo DNA methylation during monkey pre-implantation embryogenesis
Source: Cell Res. 2017 Feb 24;27(4):526–39. doi: 10.1038/cr.2017.25 (PMC5385613; doi:10.1038/cr.2017.25)
Supplement: Supplementary information, Figure S1 — The sensitivity and reproducibility of T-WGBS technique. [file cr201725x1.pdf]

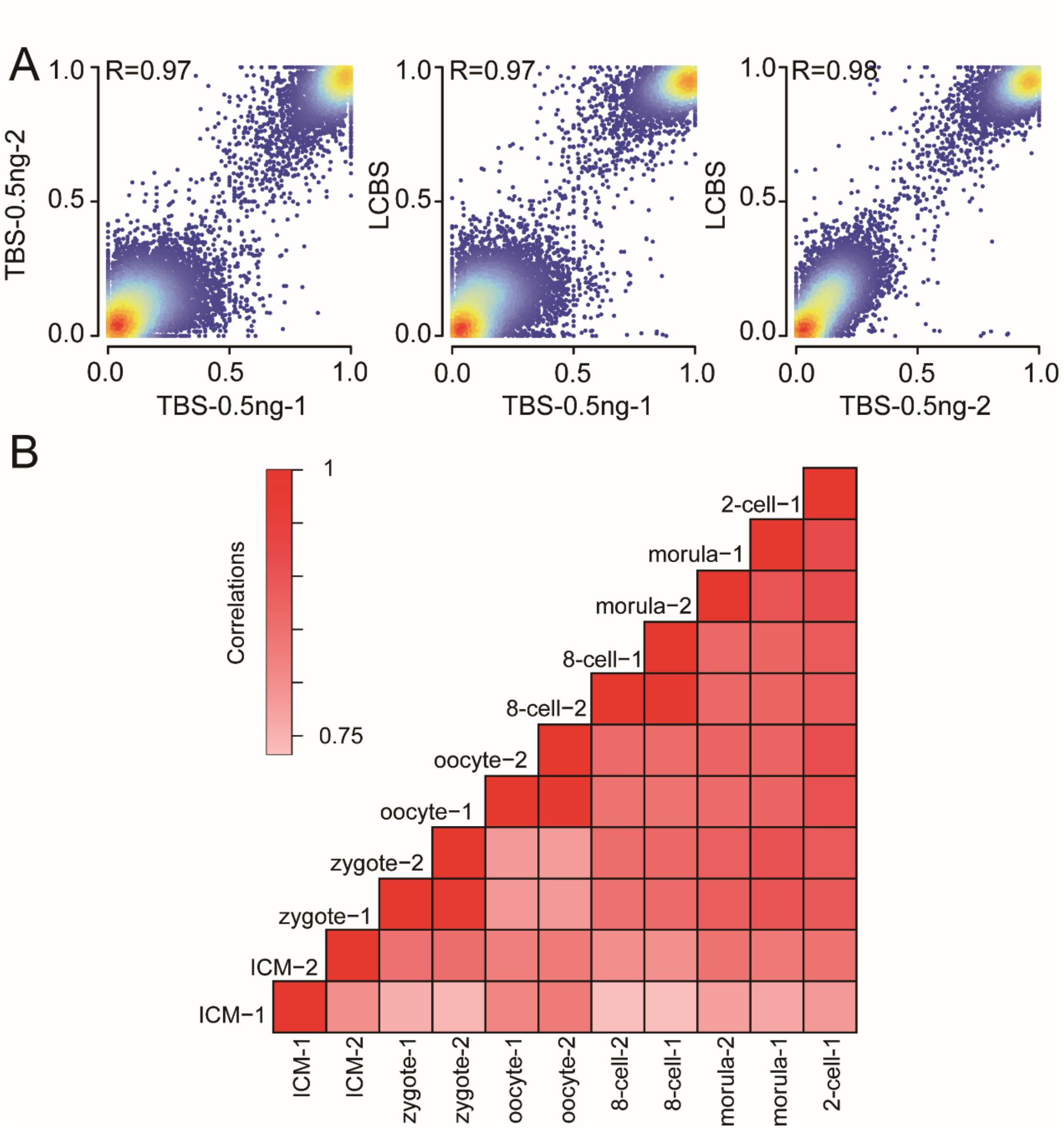

Supplementary Figure S1 The sensitivity and reproducibility of T-WGBS technique. (**A**) Comparison between standard WGBS (marked as LCBS) and the modified T-WGBS (marked as TBS). Smooth scatterplots of methylation levels of cytosines in CG contexts with sequencing depth >=20× from two replicates of new T-WGBS libraries from 0.5ng initial DNA input, as exemplified by rice chromosome 12 (left panel). Smooth scatterplots of mean methylation levels of promoter regions in CG contexts with depth >=5× in both LCBS (Y-axis) and T-WGBS (X-axis), as exemplified by rice chromosome 12 (middle and right panel). (**B**) Pearson correlation heatmap among the methylomes of T-WGBS samples. The color key from white to red indicates low to high correlation, respectively.
