# Supplementary material for: De novo DNA methylation during monkey pre-implantation embryogenesis
Source: Cell Res. 2017 Feb 24;27(4):526–39. doi: 10.1038/cr.2017.25 (PMC5385613; doi:10.1038/cr.2017.25)
Supplement: Supplementary information, Figure S2 — Isolation of monkey embryonic samples for T-WGBS. [file cr201725x2.pdf]

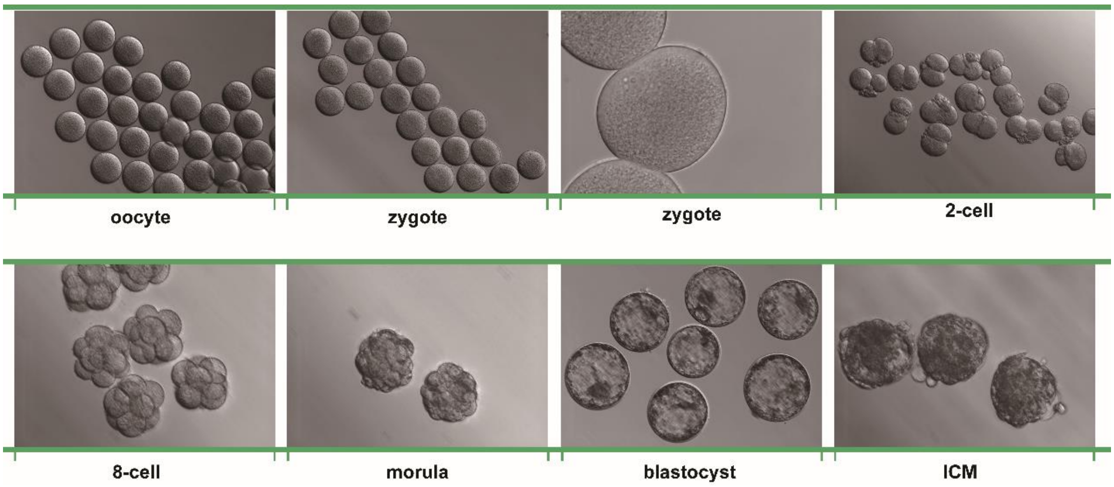

Supplementary Figure S2 Isolation of monkey embryonic samples for T-WGBS. Images of embryos that were harvested for analyses are shown. Polar bodies were removed by microdissection during collection of oocytes and zygotes. For later stages, polar bodies were removed at zygote stage and kept on culturing until sample collections. Zona pellucidae of embryos and oocytes were removed by brief exposure (45-60s) to 0.5% pronase in the culture medium. At zygote stage, during harvesting, paternal and maternal pronuclear had not fused yet.
