# Supplementary material for: De novo DNA methylation during monkey pre-implantation embryogenesis
Source: Cell Res. 2017 Feb 24;27(4):526–39. doi: 10.1038/cr.2017.25 (PMC5385613; doi:10.1038/cr.2017.25)
Supplement: Supplementary information, Figure S3 — Global patterns of DNA methylation during monkey early embryogenesis. [file cr201725x3.pdf]

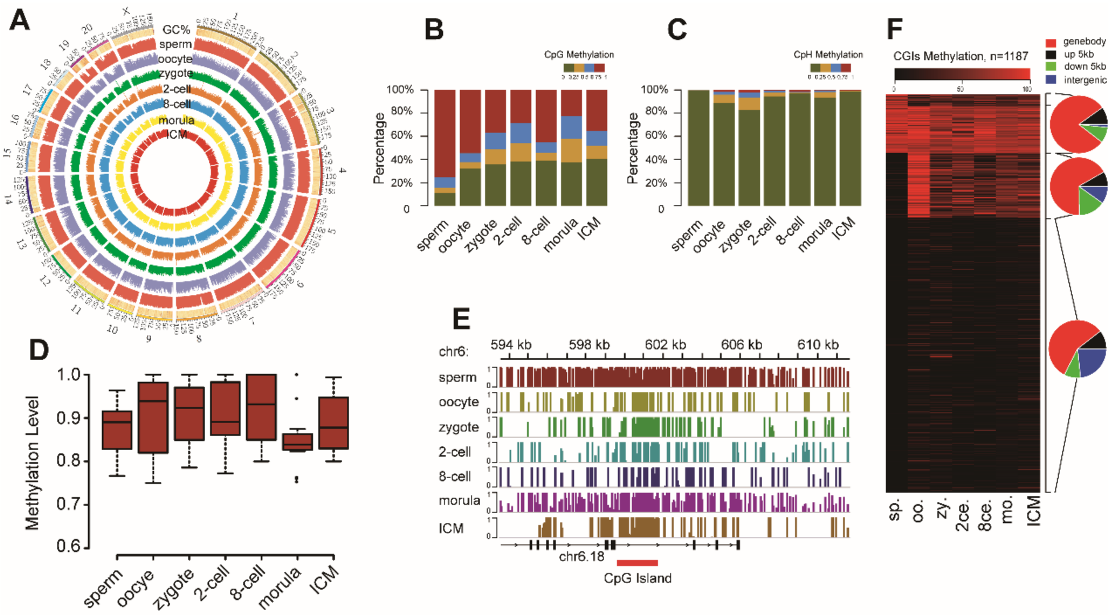

Supplementary Figure S3 Global patterns of DNA methylation during monkey early embryogenesis. (**A**) Circular representation (circus plot) of the genome-wide distribution of CpG methylation levels in gametes (sperm, oocytes) as well as embryos from zygotes to ICM. Distribution of CpG (**B**) and CpH (**C**) sites with high (≥0.75, red), intermediate (>0.25 and <0.75, yellow and blue) and low (≤0.25, green) methylation values across the genome. Note that although CpH sites generally remain hypomethylated, subtle changes in DNA methylation still occur. (**D**) Box plot of methylation levels of CGI sites that remain hypermethylated throughout the entire pre-implantation period. (**E**) Graphical representation of a representative genomic region with hypermethylated CGIs, red bar highlights a CGI in an intronic region. (**F**) Genomic distribution of CGIs across different developmental stages.
