# Supplementary material for: De novo DNA methylation during monkey pre-implantation embryogenesis
Source: Cell Res. 2017 Feb 24;27(4):526–39. doi: 10.1038/cr.2017.25 (PMC5385613; doi:10.1038/cr.2017.25)
Supplement: Supplementary information, Figure S4 — DNA methylation patterns in monkey embryos detectedby immunostaining. [file cr201725x4.pdf]

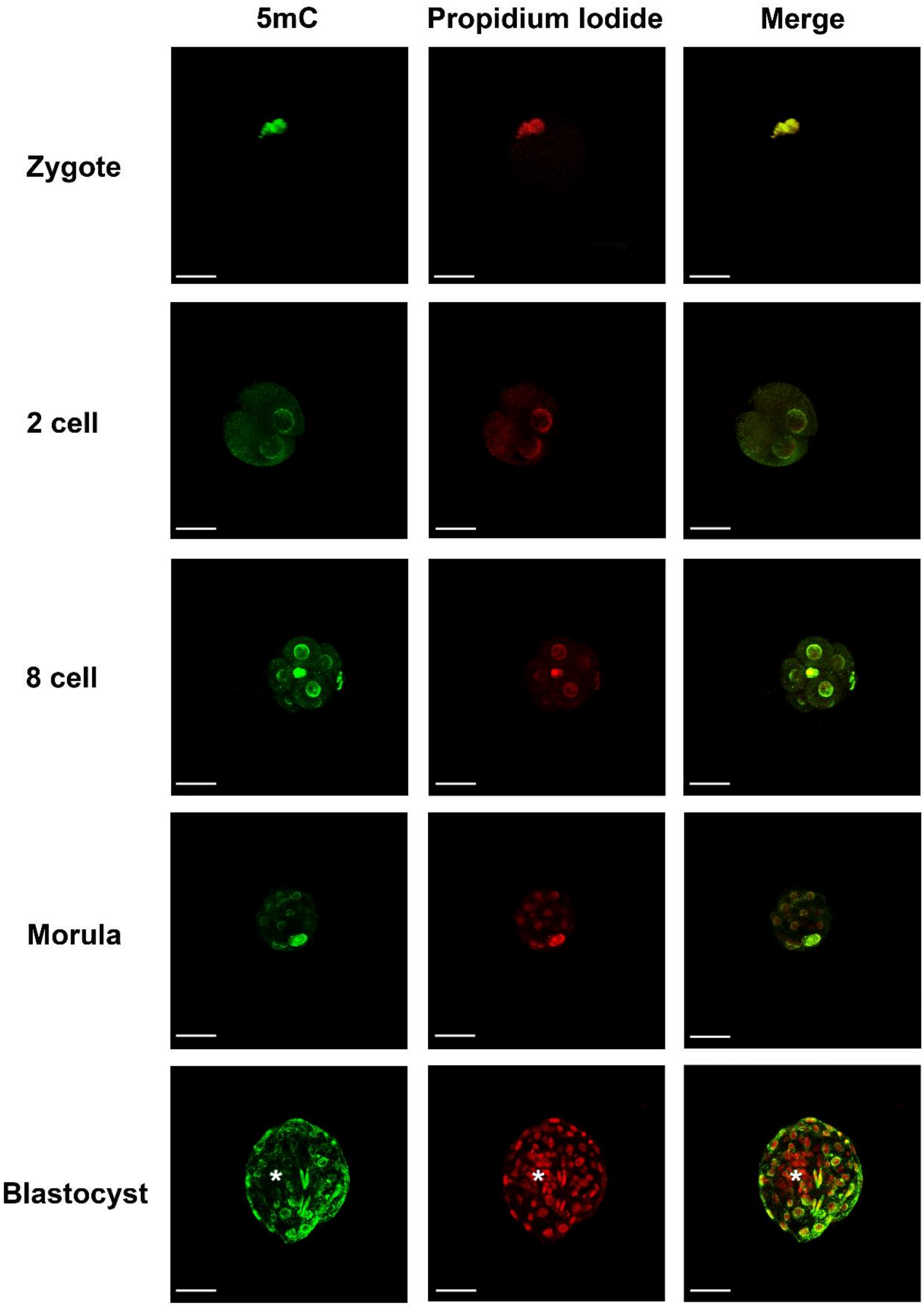

Supplementary Figure S4 DNA methylation patterns in monkey embryos detected by immunostaining. The immunostaining of 5mC from zygote to blastocyst stage. * indicates ICM cells. Note that immunostaining intensities of 5mC increased at 8-cell stage, and trophectoderm cells were with more brightly staining comparing to that of ICM cells.
