# Supplementary material for: De novo DNA methylation during monkey pre-implantation embryogenesis
Source: Cell Res. 2017 Feb 24;27(4):526–39. doi: 10.1038/cr.2017.25 (PMC5385613; doi:10.1038/cr.2017.25)
Supplement: Supplementary information, Figure S5 — DNA methylation dynamics in different genomic elements. [file cr201725x5.pdf]

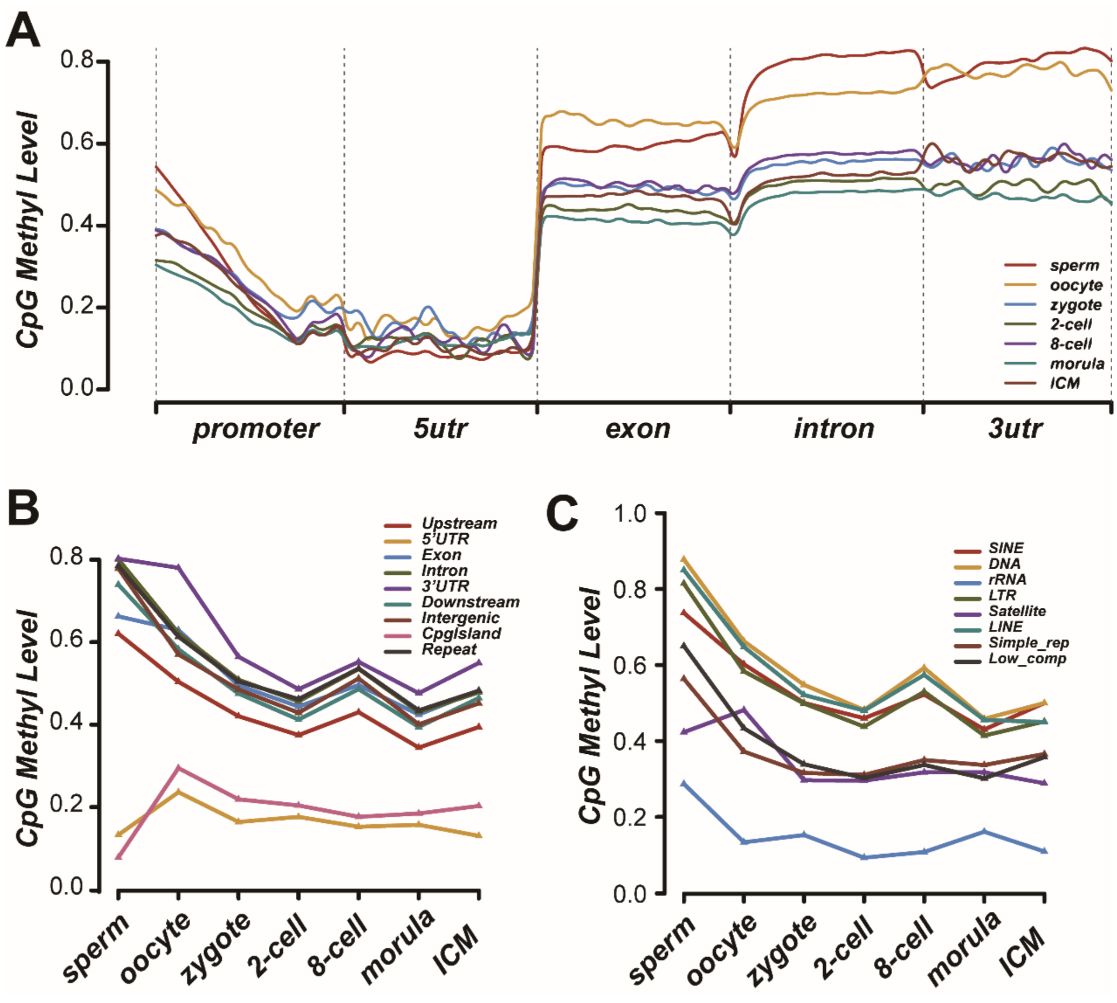

Supplementary Figure S5 DNA methylation dynamics in different genomic elements. (**A**) Levels of CpG methylation corresponding to specific gene structures and across all examined samples. (**B**) The CpG methylation dynamics of different genomic elements during monkey early embryogenesis. (**C**) The CpG methylation dynamics of different repeat elements.
