# Supplementary material for: De novo DNA methylation during monkey pre-implantation embryogenesis
Source: Cell Res. 2017 Feb 24;27(4):526–39. doi: 10.1038/cr.2017.25 (PMC5385613; doi:10.1038/cr.2017.25)
Supplement: Supplementary information, Figure S6 — Sequential pairwise comparisons between gametes and all pre-implantation stages. [file cr201725x6.pdf]

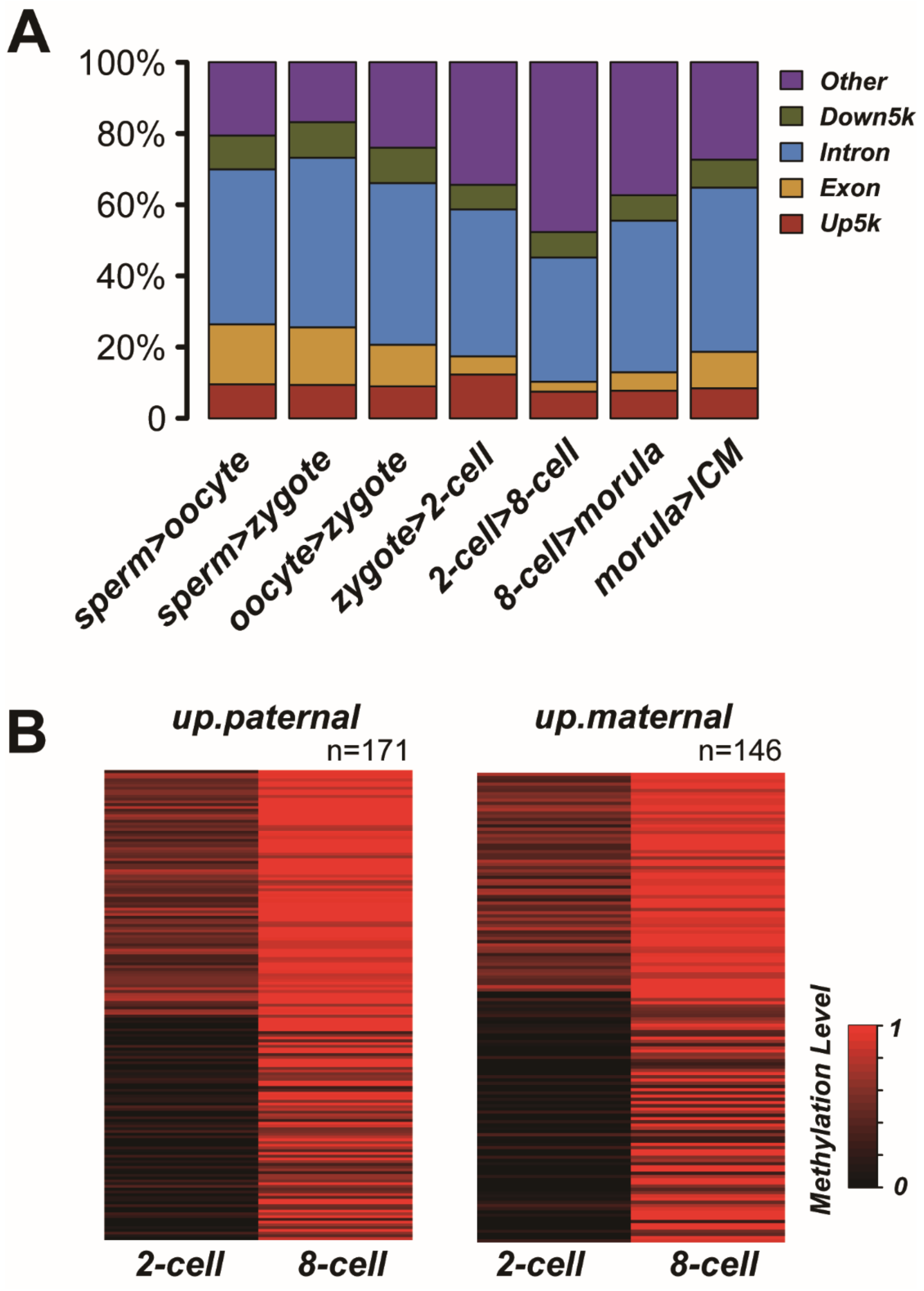

Supplementary Figure S6 Sequential pairwise comparisons between gametes and all pre-implantation stages. (**A**) Distributions of CpG DMRs corresponding to gene structures. (**B**) *de novo* CpG methylation during 2-cell to 8-cell stage transition happens evenly at both paternal and maternal alleles.
