# Supplementary material for: De novo DNA methylation during monkey pre-implantation embryogenesis
Source: Cell Res. 2017 Feb 24;27(4):526–39. doi: 10.1038/cr.2017.25 (PMC5385613; doi:10.1038/cr.2017.25)
Supplement: Supplementary information, Figure S7 — de novo DNA methylation across all stages of early human embryogenesis. [file cr201725x7.pdf]

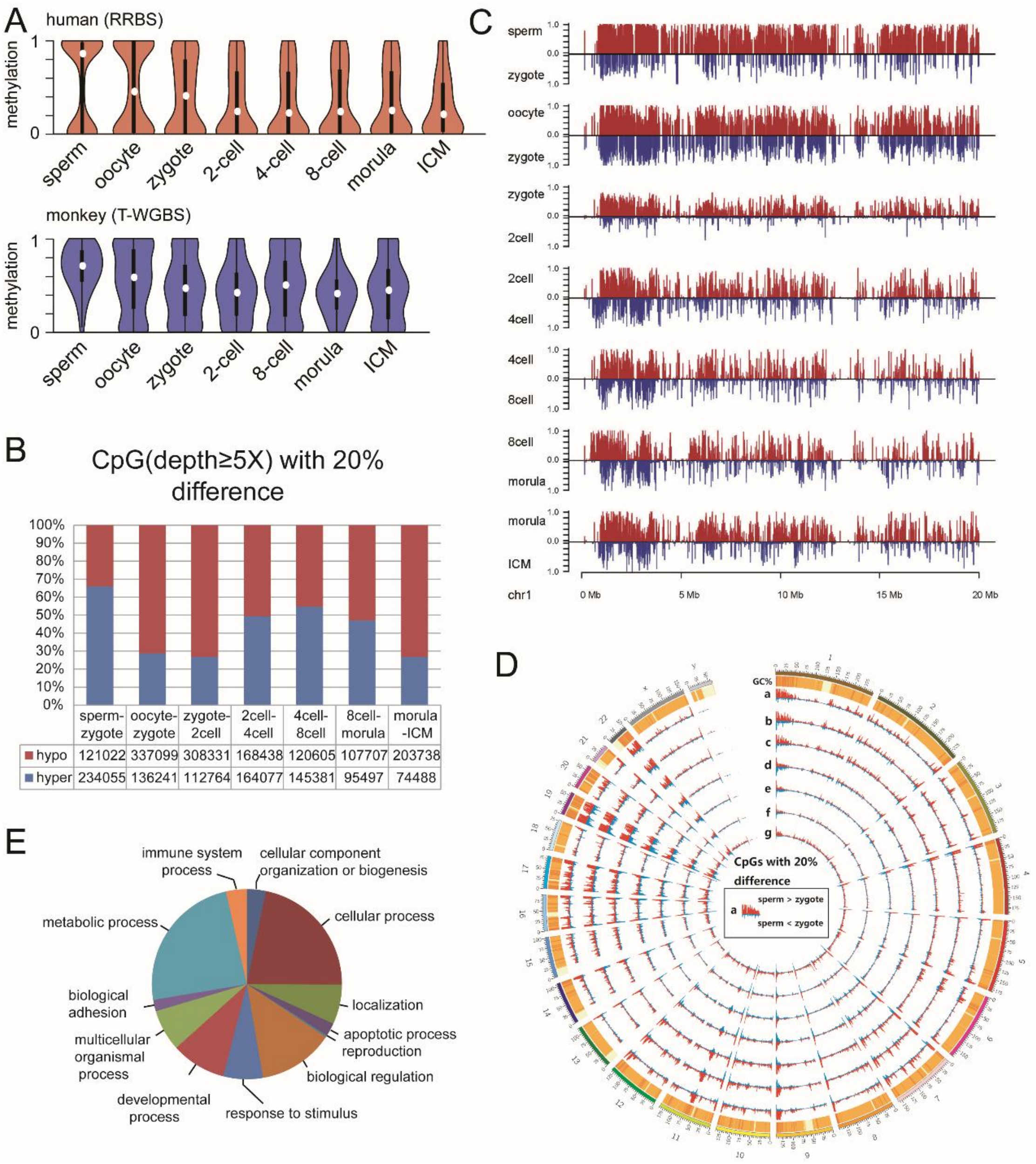

Supplementary Figure S7 *de novo* DNA methylation across all stages of early human embryogenesis. (**A**) Averaged DNA methylation levels of each stage of human and monkey pre-implantation embryos at CpG sites. (**B**) Histogram of the CpGs (depth>=5X) with 20% difference between consecutive stages. (**C**) Graphical representation of the CpGs (depth>=5X, with 20% difference) distribution at a representative genomic region of chromosome 1 between human consecutive pre-implantation stages. (**D**) Circos plot of the genome-wide distribution of CpGs (depth>=5X, with 20% difference) across all sequential pairwise comparison. Circle a, b, c, d, e, f and g represent comparison between sperm and zygote (a), oocyte between zygote (b), zygote between human 2-cell stage embryos (c), 2-cell between 4-cell stage embryos (d), 4-cell between 8-cell stage embryos (e), 8-cell between morula stage embryos (f) and morula between ICM (g). Red lines represent former hypermethylated CpGs, and blue lines represent latter hypermethylated CpGs. (**E**) Pie chart represents functional classification of biological process of genes with hypermethylated promoters at 8-cell stage.
