# Supplementary material for: De novo DNA methylation during monkey pre-implantation embryogenesis
Source: Cell Res. 2017 Feb 24;27(4):526–39. doi: 10.1038/cr.2017.25 (PMC5385613; doi:10.1038/cr.2017.25)
Supplement: Supplementary information, Figure S8 — Methylation dynamics of paternal and maternal specific loci in X chromosomeduring early embryogenesis. [file cr201725x8.pdf]

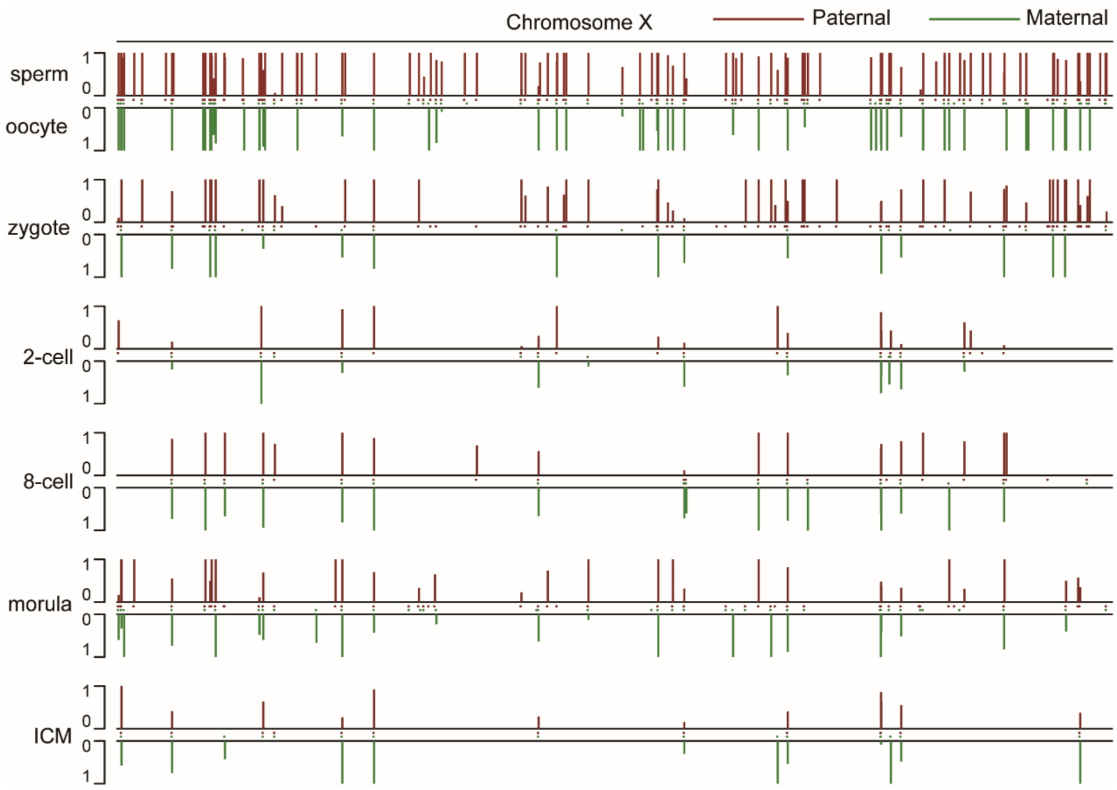

Supplementary Figure S8 Methylation dynamics of paternal and maternal specific loci in X chromosome during early embryogenesis. Paternal specific sites (dots under the line) are labeled in maroon and maternal sites in green. Note that the DNA methylation dynamics of the X chromosome closely resemble genome-scale dynamics. No obvious X-specific DNA methylation changes were detected (CpG sites on X chromosome, with 5×depth coverage were shown).
