# Supplementary material for: De novo DNA methylation during monkey pre-implantation embryogenesis
Source: Cell Res. 2017 Feb 24;27(4):526–39. doi: 10.1038/cr.2017.25 (PMC5385613; doi:10.1038/cr.2017.25)
Supplement: Supplementary information, Table S1 — Summary of the sequencing qualities, the covered CpG sites and their meancoverage depths at 1×, 5× in each embryonic stage. [file cr201725x9.pdf]

Supplementary Table S1. Summary of the sequencing qualities, the covered CpG sites and their mean coverage depths at 1×, 5× in each embryonic stage.

Sample	C-type	Genome Depths	Effective Depths	Number of Cytosine(1X)	Cytosine covered(1X)	Number of Cytosine(5X)	Cytosine covered(5X)	Bisulfite conversion
Sperm	CpG	11.57	11.92	49,781,853	97.07%	41,651,354	81.22%	99.91%
	CpH	11.58	11.81	974,725,238	98.06%	794,537,615	81.55%	
Oocyte	CpG	9.49	27.28	17,688,372	34.49%	9,011,695	17.57%	96.66%
	CpH	4.76	13.10	365,765,476	36.70%	193,881,561	19.51%	
Zygote	CpG	5.05	13.60	18,981,707	37.01%	6,913,067	13.48%	94.22%
	CpH	2.89	7.58	377,244,668	38.06%	138,398,970	14.09%	
2 cell	CpG	4.95	9.83	25,749,944	50.21%	10,844,144	21.15%	95.84%
	CpH	3.76	7.10	530,330,163	53.34%	229,896,909	23.24%	
8 cell	CpG	5.28	20.11	13,478,189	26.28%	9,574,456	18.67%	96.24%
	CpH	4.57	17.69	258,789,106	26.01%	195,454,305	19.64%	
Morula	CpG	9.55	12.35	39,734,062	77.48%	27,462,001	53.55%	96.05%
	CpH	9.31	11.73	790,403,756	79.59%	551,967,815	55.90%	
ICM	CpG	3.88	10.87	18,346,113	35.77%	10,540,580	20.55%	98.01%
	CpH	3.70	10.68	339,571,683	34.50%	194,135,115	19.78%	
